# Initial assessment of a novel smoking cessation program integrating app-based behavioral therapy and an electronic cigarette: results of a pilot study

**DOI:** 10.1186/s13722-025-00559-w

**Published:** 2025-03-27

**Authors:** Helen Schiek, Tobias Esch, Cosima Hoetger

**Affiliations:** Institute for Integrative Health Care and Health Promotion (IGVF), Faculty of Health/School of Medicine, Witten/Herdecke University, Witten, Germany

**Keywords:** Smoking cessation, Digital health, MHealth, Cognitive behavioral therapy, Mindfulness, Electronic cigarettes

## Abstract

**Background:**

Detrimental smoking-related health outcomes warrant the investigation of novel smoking cessation interventions; the cessation program nuumi integrates digital behavioral therapy and an electronic cigarette (EC).

**Objective:**

The relationship between program participation and smoking cessation among adults who smoke and are motivated to quit was investigated, as well as program acceptability, changes in smoking-related outcomes, including cigarettes per day (CPD), urges to smoke and psychophysiological health variables (perceived stress, mindfulness, cessation-related self-efficacy, life satisfaction, subjective psychophysiological health) and their associations with smoking cessation.

**Methods:**

A prospective 6-month single-arm pilot study was conducted; 71 adults who smoked and were motivated to quit received a cognitive behavioral therapy (CBT) app, a closed-system EC, and pods containing decreasing nicotine concentrations. Online surveys were issued at baseline, and at 4, 8, 12, and 24 weeks post-baseline. Intention-to-treat (ITT) and complete-case analyses were conducted to assess self-reported 7-day point prevalence of smoking abstinence (PPA; primary outcome), 30-day PPA, and repeated PPA. T-tests and logistic regressions were used to assess changes in secondary outcomes CPD, urges to smoke, and psychophysiological health variables by smoking status at 12 and 24 weeks, and their relationship with cessation.

**Results:**

Per ITT, self-reported abstinence rates were high at 12 weeks (39.4%), and 24 weeks (32.4%), as was 30-day PPA of 32.4% at both 12 and 24 weeks. Repeated PPA per ITT was 22.5% at both 12 and 24 weeks. Non-abstinent participants significantly reduced their CPD at 12 weeks (*t*(34) = 6.12, *p* < 0.001), and at 24 weeks (*t*(30) = 6.38, *p* < 0.001). Urges to smoke and perceived stress decreased, and mindfulness, cessation-related self-efficacy, life satisfaction and subjective psychophysiological health increased significantly (all *p*s < 0.05), predominantly in individuals who reported abstinence. Lower urges to smoke, lower perceived stress, and higher self-efficacy and subjective mental health were related to greater odds of cessation at 24 weeks (all *p*s < 0.05). Most participants rated the program as highly (43%) or moderately (54%) acceptable.

**Discussion:**

Program participation seems to support cessation and improvements in smoking-related outcomes, but adjustments to the program may be needed to improve engagement and acceptability. Findings may inform the development of future trials and cessation programs.

**Trial Registration:**

German Clinical Trials Register DRKS00032652, registered prospectively 09/15/2023, https://drks.de/search/de/trial/DRKS00032652

**Supplementary Information:**

The online version contains supplementary material available at 10.1186/s13722-025-00559-w.

## Introduction

Smoking tobacco continues to be a major contributor to preventable diseases and deaths worldwide [1], and places financial strain on economies across the world [2]. Quitting smoking is associated with improvements in several psychophysiological health outcomes [3, 4], and plays an important role in reducing healthcare costs [4], and improving individuals’ quality of life [5]. Smoking cessation treatments such as nicotine replacement therapy (NRT), pharmacotherapies, and behavioral interventions have been shown to be effective in supporting adults to quit smoking [6]. However, utilization rates of such interventions remain low [7, 8], which may be partially explained by previous research findings highlighting that individuals who smoke often perceive these interventions as ineffective [9], and that these treatments not fully address the sensory, behavioral, and social aspects of smoking [10]. Electronic cigarettes (ECs) may present an alternative that addresses the aforementioned factors. ECs consist of a reservoir containing a liquid solution, a power supply, and a heating component; the liquid solution contains solvents such as vegetable glycerin and/or propylene glycol, flavorings, and may contain nicotine [11]. Despite being used by adults who smoke for cessation purposes in the European Union [12] and the United States [13], ECs are not approved as smoking cessation aids in these countries. The use of ECs is associated with significantly greater smoking abstinence rates relative to quit attempts made without the use of evidence-based cessation methods such as behavioral counseling or NRT [14], and a recent Cochrane review suggests that nicotine-containing ECs support smoking cessation more effectively than NRT [15]. A possible explanation for these findings may be that ECs may reduce withdrawal symptoms more effectively than NRT [16], and that ECs mimic the sensory and motoric experience of smoking cigarettes such as inhalation, “throat hit”, and the production of vapor clouds when exhaling [15, 17]. For some ECs, customization of device and liquid solution is possible, allowing users to decrease nicotine concentrations over time [16]. Reducing nicotine content in combustible cigarettes has been shown to reduce individuals’ desire for smoking, which has been hypothesized to be due to the reduction of smoking-related reinforcing effects, a key marker of combustible cigarettes’ dependence potential [18]. However, EC-supported cessation attempts are often accompanied by prolonged EC use post-cessation [19], and while some evidence suggests that ECs may be less harmful than combustible cigarettes in the short term, effects of long-term EC use are unclear [20], necessitating strategies to wean users off ECs after they have quit smoking.

While ECs can aid smoking cessation, evidence from trials investigating pharmacological interventions suggests that smoking cessation efforts are more effective when they are combined with behavioral support [21] such as cognitive behavior therapy (CBT) [22]. CBT can support individuals who smoke in their attempts to quit smoking by modifying smoking-related thoughts, beliefs, and behaviors, helping them improve their problem-solving and coping skills, such as cognitive restructuring of maladaptive thoughts [23]. Mindfulness-informed interventions (MIIs) are increasingly incorporated in CBT treatments and usually include training of attention regulation, body awareness, emotion regulation, and self-awareness [24]. In smoking cessation, mindfulness training teaches individuals to observe their cravings and monitor their emotional states in response to their cravings without resorting to smoking [25]. Mindfulness training may weaken the link between cravings and smoking [26, 27], and enhances individuals’ self-efficacy when it comes to regulating their emotions while abstaining from cigarettes [28]. Findings on the effect of mindfulness interventions on smoking cessation are mixed; while some systematic reviews and meta-analyses found mindfulness-based interventions to be more effective than non-mindfulness-based interventions of similar intensity, less intensity, or no treatment [29, 30], other reviews were not able to confirm these findings [31, 32].

While incorporating behavioral components into smoking cessation programs can improve their effectiveness, in-person counseling can be expensive and challenging to access for individuals willing to quit smoking with limited time or financial resources. Digital behavioral therapies such as mobile health (mHealth) apps provide an accessible, cost-effective, and scalable alternative to in-person support while still providing real-time support and personalization [33, 34]. Also, app-based interventions may promote support-seeking among individuals who are reluctant to pursue face-to-face treatment due to perceived stigma [35]. A growing body of literature highlights the effectiveness of digital CBT-based programs for smoking cessation [36, 37], and MIIs may also be effective when delivered digitally [38, 39].

However, while an increasing number of apps are designed to be used in conjunction with NRT, to our knowledge, there are no smoking cessation programs available that incorporate an app and an EC. Sanos Group GmbH (Berlin, Germany) developed a smoking cessation program integrating an EC and app-based behavioral therapy, *nuumi*. This reported trial was the first to evaluate this particular intervention.

### Objectives

This primary objective of this trial was to evaluate the relationship between participation in the nuumi program and smoking cessation among adults who smoke and who are motivated to quit by measuring self-reported 7-day point prevalence abstinence (PPA) from smoking at 12 and 24 weeks after program initiation. Secondary objectives were to examine reduction in cigarettes per day (CPD), 30-day and repeated PPA, and sample-level and within-group changes in urges to smoke, as well as in smoking-related psychophysiological health outcomes (perceived stress, mindfulness, self-efficacy to abstain from smoking, subjective health, life satisfaction). We also aimed to investigate the respective associations of the aforementioned variables with the likelihood of successful cessation. Another secondary objective was to assess the acceptability of the nuumi program.

## Methods

### Study design and setting

This prospective 6-month pilot study conducted in Germany evaluated smoking cessation outcomes, acceptability, and psychophysiological health outcomes of an mHealth intervention (nuumi). Study methods have been described elsewhere in more detail [40]. Ethics approval was obtained from the Institutional Review Board (IRB) of Witten/Herdecke University in September 2023 (123/2023). Single-arm pilot studies are commonly used to test new interventions as they allow to assess feasibility, acceptability, and initial effectiveness before advancing to more resource-intensive randomized controlled trials (RCTs) [41, 42]. This approach enabled the study to gather preliminary data on participant adherence, identify potential challenges in study procedures, and gain insight on program participation to help refine the program.

### Participants

Individuals aged 18–65 years who reported having smoked at least 5 cigarettes per day for at least one year, were motivated to quit (Motivation To Stop Scale (MTSS; [43]) > 4 points), had access to a smartphone (iOS 15/Android 11+), resided in Germany, had email access, and could read and write in German were eligible. Exclusion criteria included current pregnancy or breastfeeding, allergy to glycerin or propylene glycol, drug and/or alcohol dependence, severe psychiatric or physical illness, an illness contraindicating use of ECs, medications that could affect study outcomes (bupropion, nortriptyline, varenicline, cytisine, clonidine, antidepressants), surgery (with general anesthesia) in the last 6 weeks, use of EC/tobacco heaters/alternative tobacco products/NRT for more than 5 days during the last 30 days, and inability to consent.

### Recruitment

Target sample size estimation was based on single-arm pilot studies evaluating interventions for smoking cessation [41, 44–46]. In order to address the primary and secondary aims of this trial, and to account for attrition, the target sample size was 70 participants. A total of 77 participants were recruited between October 2023 and January 2024 via online advertisements, flyers, and a study website. Interested individuals were prompted to register using the study website and complete a screening survey. Eligible participants were sent an online consent form to sign. After submitting the signed form, participants were emailed a link to the baseline survey (t_0_). To prevent duplicate entries, research staff screened names and email addresses. Each participant received a personalized email with an individual access key to the surveys. An incentive of €10 for each completed survey (4-, 8-, 12-, 24-week follow-ups) was paid to the participants once the trial had ended.

### Study timeline and data collection

Over a period of six months, participants were asked to respond to five online surveys administered at baseline (t_0_), 4 weeks (t_1_), 8 weeks (t_2_), 12 weeks (t_3_), and 24 weeks (t_4_) post-baseline. After basleline, participants received access to the nuumi intervention. Participants were granted two weeks to complete each survey. Primary and secondary outcomes were analyzed using data from the 12-week and 24-week follow-ups. Data were collected using the web-based tool LimeSurvey (LimeSurvey GmbH, Hamburg, Germany). Participants received a personalized survey link via email and up to two reminder emails over two weeks to encourage completion. Participants could skip questions if they wanted to. Each survey included an optional text box for feedback or comments. Following the baseline survey, participants were instructed to download the nuumi app and received a voucher to order the EC and pods from the manufacturer's website at no charge. Total program cost differed between participants as the number of pods provided varied depending on the number of CPD reported at t_0_; however, all costs associated with ordering the program were covered by the voucher.

**Fig. 1:**
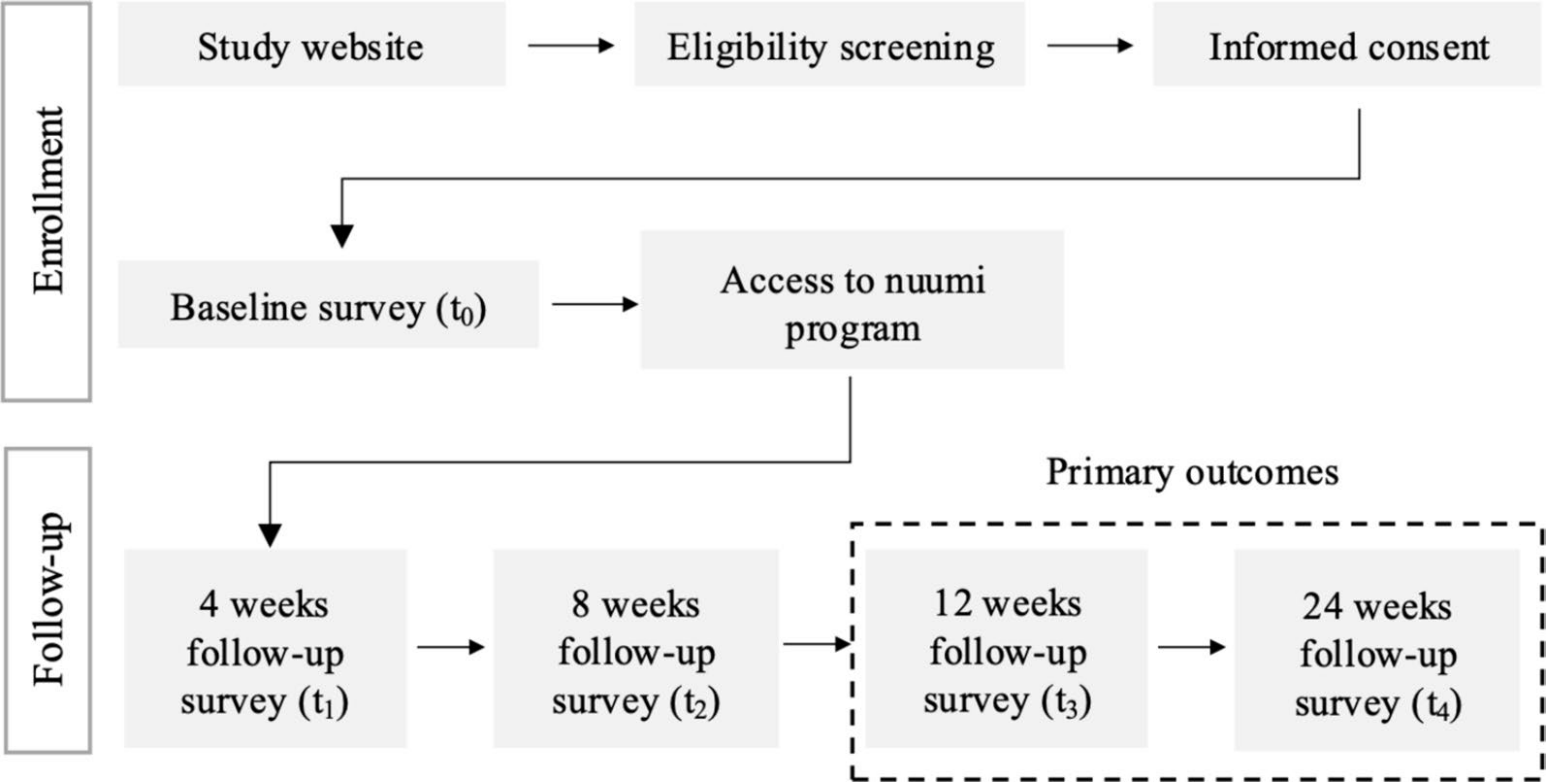
Participant flow and study design

Figure 1 shows participant flow and study design in an adapted Consolidated Standards for Reporting Trials (CONSORT-EHEALTH) diagram for pilot and feasibility trials [47]. Data were collected using the web-based tool LimeSurvey (LimeSurvey GmbH, Hamburg, Germany). Participants received a personalized survey link via email and up to two reminder emails over two weeks to encourage completion. Participants could skip questions if they wanted to. Each survey included an optional text box for feedback or comments.

### Intervention

Nuumi is a digital therapeutic intervention featuring app-based behavioral therapy and an EC. The intervention and its theoretical foundation have been described in detail in the study protocol [40], and in a previous feature-level analysis of the nuumi program [48]. Initially, participants were instructed to use the EC to replace cigarettes whenever they experienced cigarette cravings. Participants were not required to quit smoking immediately; they were advised to switch from cigarettes to the EC gradually over a 2-week period or set a quit date. Simultaneously, participants were asked to use the app for behavioral support.

#### EC component

**Fig. 2:**
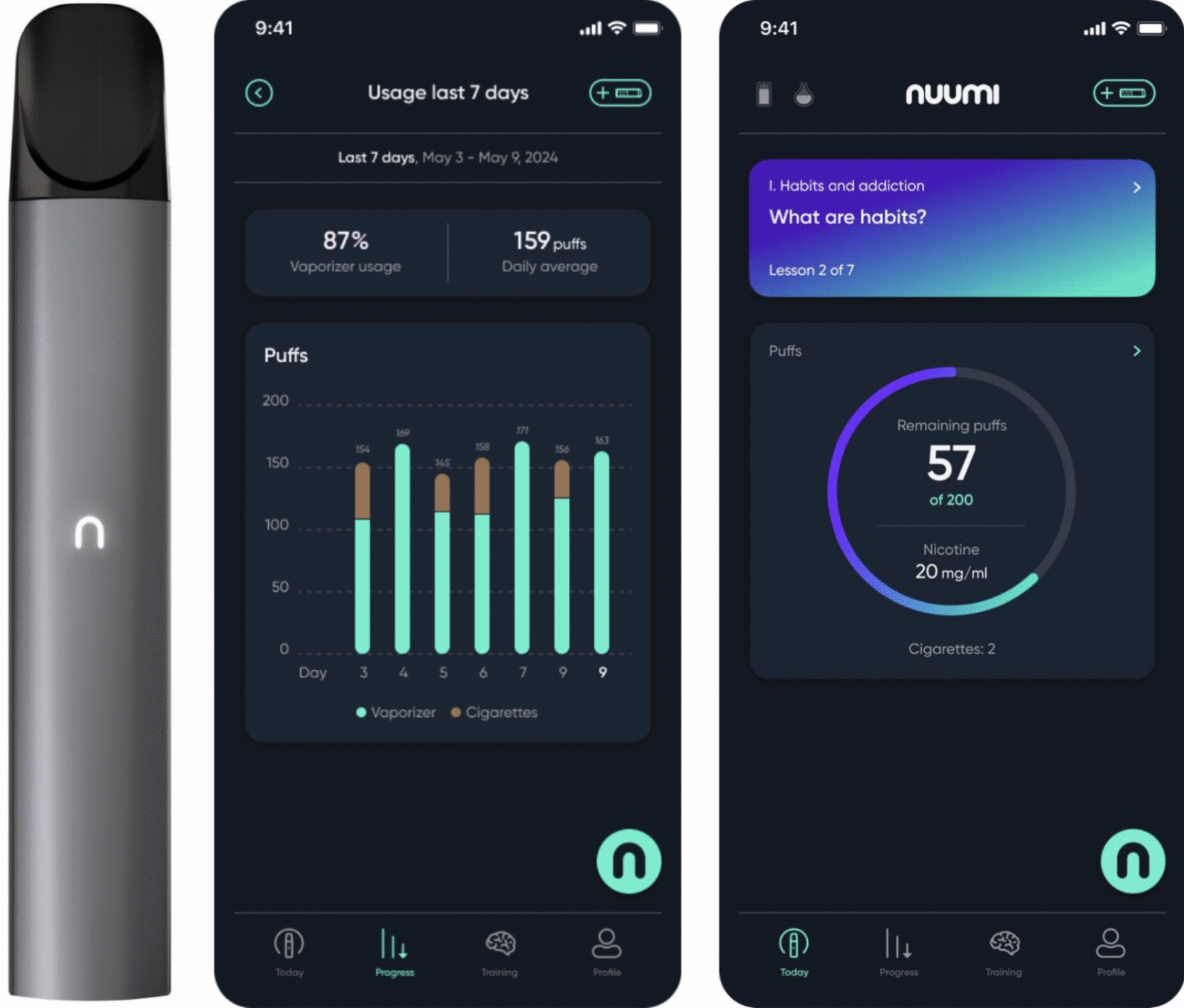
(1) nuumi EC, (2) app section *Today* depicting current behavioral therapy module, daily puffs, puff budget, nicotine strength of current pod, and tracked cigarettes, (3) app section *Progress* depicting EC puffs and cigarette consumption statistics

The EC, developed and provided to participants by the study's funder, Sanos Group GmbH, was a closed system device with non-refillable pods, which had to be activated via the app connected to the EC via Bluetooth. The liquid solution in the pods included propylene glycol, glycerol, nicotine, and flavoring. The EC was powered by a 450 mAh battery, and activated by taking a puff; settings were not modifiable. Participants received a kit containing the EC, charger, power bank, manuals, and pods. Participants could choose from two tobacco flavors (“Tobacco No. 1” and “Red Galliant”) differing from one another in tobacco flavor intensity, and received a number of pods tailored to their initial cigarette consumption ranging in nicotine strength from 20 to 0 mg/ml, decreasing in 2 mg/ml increments. Participants were provided with an average of 65 pods, with the specific number of pods depending on the number of CPD reported at baseline. The provided number of pods was calculated to last for around 16 weeks based on a guideline by the manufacturer. The manufacturer’s use recommendations were part of the baseline survey and could also be accessed via the app. Instructions included starting with a nicotine concentration of 20 mg/ml and gradually transitioning to pods with lower nicotine concentrations. Each time a pod required replacement, the app provided guidance on what nicotine concentration to use next. The Bluetooth connection between EC and the nuumi app allowed participants to track their daily puffing patterns (see Fig. 2). After two weeks, to prevent compensatory puffing [42], participants were issued a daily puff budget calculated by the manufacturer based on participants’ daily number of puffs recorded until this point. Participants were allowed to adjust their puff budgets using an app function.

#### Behavioral therapy component

**Fig. 3:**
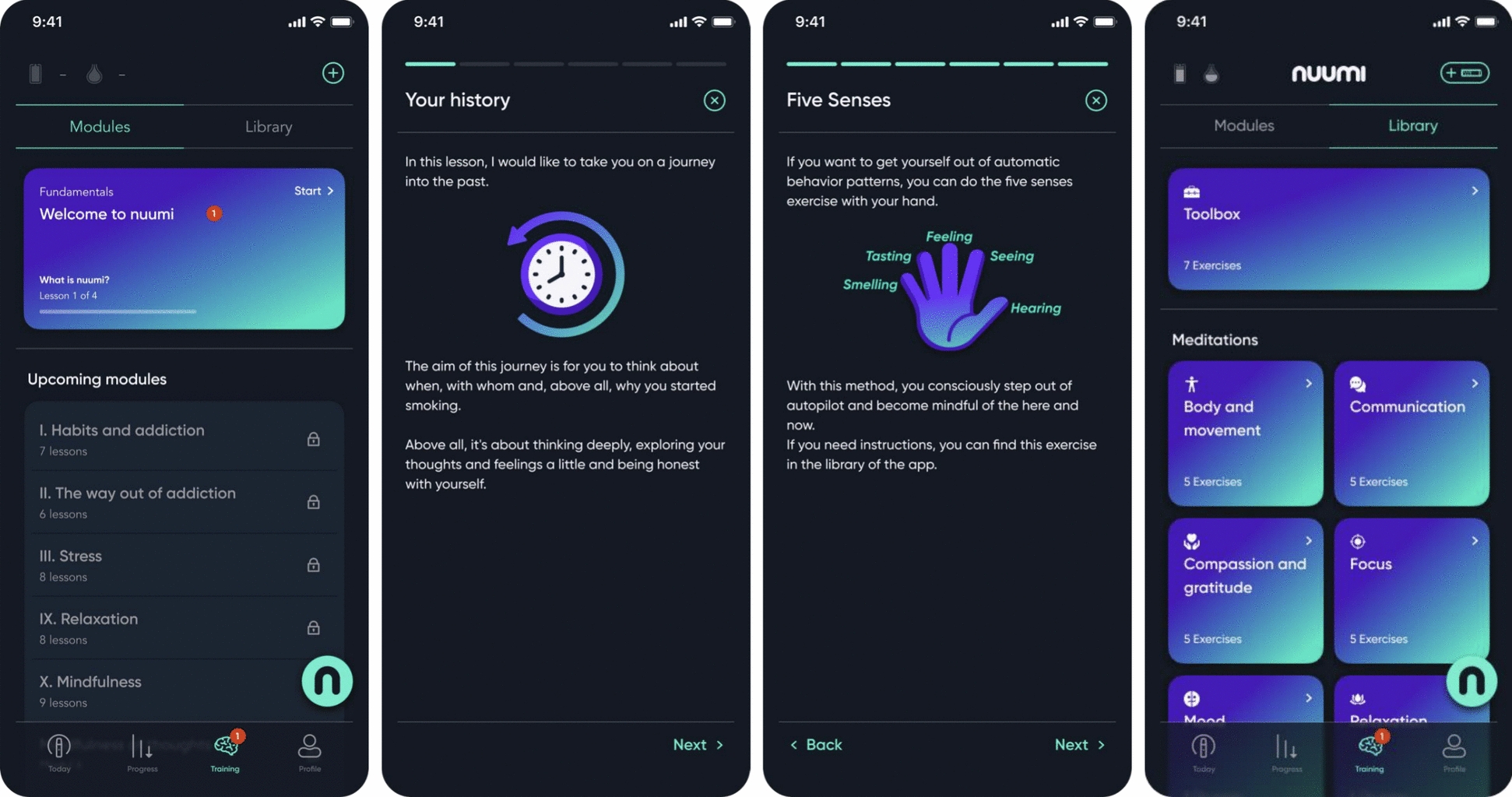
(1) Behavioral therapy modules overview, (2) and (3) examples of lessons within the modules, (4) library consisting of a *Toolbox* and meditation audios

Participants received behavioral support through the nuumi app to support them in transitioning from cigarettes to the EC, and to reduce and cease EC use over time with the goal of achieving abstinence of both products. The behavioral component featured evidence-based CBT-based and mindfulness-informed content. Based on an in-person health promotion and stress management course certified by the Central Prevention Testing Center (ZPP) of the German statutory health insurance, the behavioral component of the intervention covered content on behavior, exercise, relaxation, and nutrition (BERN, [49, 50]) in digitalized format. The content was delivered via audio, interactive exercises, and quizzes. Participants were asked to complete 11 modules sequentially (see Fig. 3). Daily push notifications were sent to participants, delivering text messages with motivational and educational content related to the lessons. Content of the modules has been described elsewhere in detail [40]. The app's *Toolbox* function offered abbreviated coping techniques for managing cravings, stress, negative thoughts, and emotions (e.g., "urge surfing" [51], and also included a meditation library (see Fig. 3) with 32 guided meditations accompanied by binaural beats [52].

Participants could monitor their cessation progress in the nuumi app by tracking both combustible cigarettes and EC puffs. Each cigarette was recorded as twelve puffs; cigarette puffs were added to daily EC puff counts and displayed as a single number. The app featured a dashboard displaying nicotine concentration currently in use, daily puff counts, progress in behavioral therapy modules, and meditation minutes. Self-efficacy for quitting smoking was assessed weekly (“How confident are you that you will be completely abstinent from smoking cigarettes in one year from now? not confident at all—very confident). Daily check-ins during the initial 14 days provided information on EC usage and motivational support. Daily push notifications delivered motivational messages aligned with therapy modules. App updates during the trial focused on minor bug fixes.

### Outcomes

#### Sociodemographic variables

Sociodemographic variables included age (in years), gender (male, female, diverse), and highest level of education (no professional qualification, recognized professional training, bachelor’s degree or equivalent, master’s degree or equivalent, doctorate).

#### Primary and secondary outcomes

The primary outcome was self-reported 7-day PPA from smoking cigarettes at 12 and 24 weeks, operationalized as not having smoked any cigarettes in the past 7 days. Secondary outcomes at 12 weeks and 24 weeks were reduction in CPD, 30-day PPA from smoking cigarettes, repeated PPA (self-reported 7-day PPA at multiple consecutive assessment points; i.e. repeated PPA at the 12-week follow-up was defined as self-reported 7-day PPA at the 4-, 8-, and 12-week follow-ups; repeated PPA at the 24-week follow-up was defined as self-reported 7-day PPA at the 4-, 8-, 12-, and 24-week follow-ups), and urges to smoke (Verlangen-zu-Rauchen-Skala (Urges to Smoke Scale); VRS; [52]). Additional psychophysiological health-related secondary outcomes assessed at 12 and 24 weeks included perceived stress (Perceived Stress Scale; PSS-10; [53]), mindfulness (Freiburg Mindfulness Inventory short version; FFA; [54]), smoking self-efficacy (Smoking Self-Efficacy Questionnaire; SEQ-12; [55]), life satisfaction (Kurzskala Lebenszufriedenheit-1 (Short Scale Life Satisfaction-1); L-1; [56]), and subjective health (Short Form Health Survey; SF12; raw sum scores; [57, 58]). Another secondary outcome was acceptability of the nuumi program, operationalized as (1) usefulness in quitting smoking ("How helpful do you find the program in not smoking cigarettes?", "To what extent does the program increase your confidence to quit smoking?”), (2) satisfaction with the program ("How would you rate your overall satisfaction with the smoking cessation program?" / "How likely are you to recommend the program to friends or colleagues who want to quit smoking?"), (3) informativeness of the content ("How informative did you find the content of the behavioral training?”). As another acceptability outcome, usability of the nuumi program was assessed by the System Usability Scale (SUS; [59]) at 24 weeks.

#### Other variables

##### Adherence and engagement

Adherence was operationalized as (1) self-reported use of the EC (current use and number of days of use (“Have you used the e-cigarette (vaporizer) allocated to you as part of the nuumi program within the last 7 days? [Yes/No]”, “How many days did you use the e-cigarette? [1–7]”), and (2) self-reported engagement with the app (number of daily/weekly uses, number of modules completed, minutes meditated, (“How many days a week do you use the nuumi app? [0–7], “How many behavioral training modules have you completed so far? [0–10]”, “How many minutes have you meditated in total during the behavioral training so far? [x minutes]”). Participants could retrieve the information on modules completed and minutes meditated from the app.

##### Other smoking-behavior related variables

Other smoking behavior-related variables included use of alternative tobacco products and/or ECs, current participation in other smoking cessation programs, and current use of NRT.

### Statistical analyses

Analyses were conducted using R version 4.4.0 (R Core Team, 2024). For categorical variables, frequencies and percentages were calculated, and for continuous variables, we analyzed means (M) and standard deviations (SD). For smoking cessation outcomes, intention-to-treat (ITT) analyses were conducted, assuming all participants not completing a follow-up survey had resumed smoking. In addition, complete case analyses (CCA) were conducted, i.e. only participants who completed the 12-week and/or the 24-week survey were included in these analyses. Statistical comparisons of baseline characteristics between individuals who did and did not report smoking abstinence at 12- and 24-weeks follow-up were conducted. For continuous variables (e.g., age, cigarettes per day, self-efficacy), independent-samples t-tests were conducted to compare means at baseline, and for binary variables (e.g., gender, education), chi-square tests were performed.

We used one-sample t-tests to assess whether urges to smoke and psychophysiological health outcomes changed significantly from baseline to the 12-week and 24-week follow-ups. For assessing bivariate changes in these variables from baseline to 12 and 24 weeks by smoking status (“abstinent” or “non-abstinent” participants), we conducted further t-tests.

To examine the relationship between urges to smoke (VRS) and smoking cessation, and psychophysiological health outcomes (PSS-10, FFA, SEQ-12, L-1, SF12) and smoking cessation, we conducted logistic regressions using each of the outcomes for each of the variables at 12 and 24 weeks post-baseline. The respective outcome served as a predictor variable, and 7-day PPA from smoking cigarettes served as the dependent variable. To control for multiple comparisons, we applied Benjamini-Hochberg (BH) adjustments [61]. In total, we fit 4 logistic regression models for urges to smoke and 12 for the psychophysiological health outcomes. Statistical significance was set at α = 0.05.

## Results

**Fig. 4:**
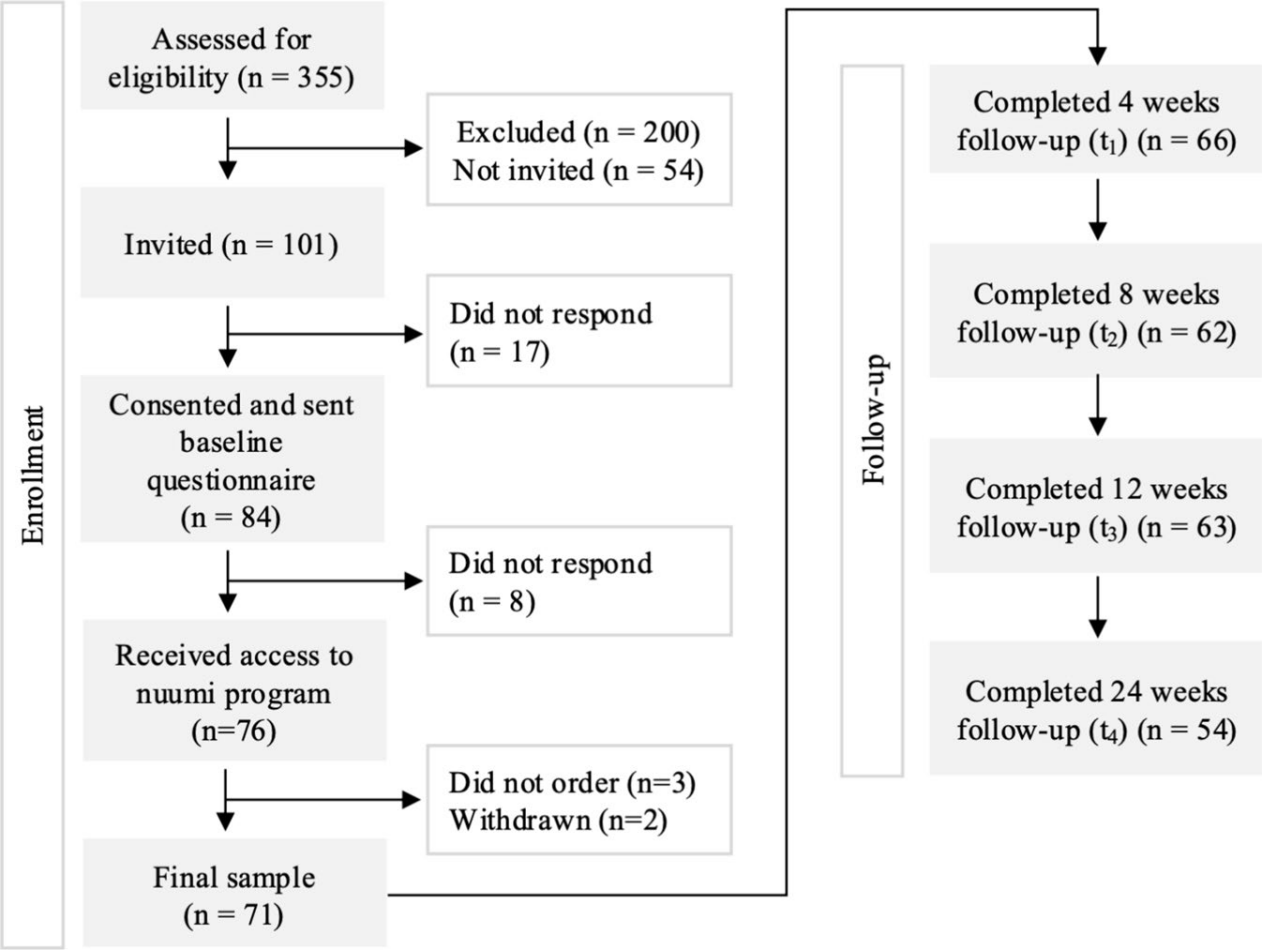
Study enrollment and attrition

Between October 2023 and January 2024, 355 interested individuals filled out the screening form; 155 individuals met the eligibility criteria. The first 87 individuals who met the inclusion criteria were contacted to fill out informed consent. Eighty-four individuals gave consent to participate and were invited to fill out the baseline survey. Seventy-six individuals completed the baseline survey. All individuals who completed the baseline survey received a voucher to order the nuumi program. Three individuals did not order the program; two individuals resigned from study participation and were subsequently excluded from the study, resulting in a final sample of 71 participants. Response rates were 93.0% (66/71) for the 4-weeks survey, 87.3% (62/71) for the 8-weeks survey, 88.7% (63/71) for the 12-weeks survey, and 76.1% (54/71) for the 24-weeks survey. Figure 4 shows study enrollment and attrition.

### Baseline participant characteristics

Participants’ age at baseline ranged from 21 to 64 years (M = 39.55, SD = 10.84); 69.0% (49/71) of the participants were women, and 41.9% (29/71) reported having earned a college degree (Bachelor’s degree or higher). On average, participants smoked 17.7 cigarettes per day (SD = 6.45, min = 6, max = 30). According to the Fagerström Test for Nicotine Dependence, 14.1% (10/71) of participants reported low levels of nicotine dependence, 28.2% (20/71) reported moderate levels of nicotine dependence, and 57.7% (41/71) reported high levels of nicotine dependence. Detailed information on baseline participant characteristics is provided in Table 1, and complete results of the baseline survey are provided in Table A 1 of Appendix A.

**Table 1:** Baseline participant characteristics

Characteristics	Statistics
Female, n (%)	49 (69.0)
Age, M (SD)	39.55 (10.8)
College degree, n (%)	
College degree	29 (41.9)
No college degree	41 (59.1)
Cigarettes per day (CPD), M (SD)	17.73 (6.45)
Years of smoking, M (SD)	21.42 (10.91)
Nicotine dependence	
M (SD)	5.34 (2.29)
Low, n (%)	10 (14.1)
Moderate, n (%)	20 (28.2)
Strong, n (%)	41 (57.8)
VRS, M (SD)	
Urge frequency	4.07 (0.64)
Urge intensity	3.73 (0.81)
PSS-10	
M (SD)	18.92 (5.01)
Low, n (%)	10 (15.1)
Moderate, n (%)	55 (77.5)
High, n (%)	6 (8.4)
FFA (M, SD)	2.57 (0.39)
SEQ-12, M (SD)	33.42 (8.90)
L-1, M (SD)	5.97 (1.67)
SF12	
SF12 PH, M (SD)	16.07 (2.22)
SF12 MH, M (SD)	18.80 (3.19)

Note*.* VRS = Urges to Smoke Scale, PSS-10 = Perceived Stress Scale, FFA = Freiburg Mindfulness Inventory, SEQ-12 = Smoking Self-Efficacy Questionnaire, L-1 = Short Scale Life Satisfaction-1, SF12 PH = Short Form Health Survey Physical Health, SF12 MH = Short Form Health Survey Mental Health

### Adherence and engagement

#### App and behavioral therapy use

Twelve weeks after program initiation, participants indicated they used the nuumi app on an average of 2.81 (SD = 2.62) days per week. On average, participants had completed 4.63 (SD = 2.81) modules of the behavioral training by the 12-week follow-up, and had meditated on average 34.85 (SD = 66.93) minutes using the app’s meditation library. AT the 24-week follow-up, participants reported using the nuumi app on an average of 0.94 (SD = 1.57) days per week. On average, participants had completed 4.89 (SD = 3.06) modules of the behavioral training, and had meditated on average 46.98 (SD = 112.79) minutes using the app’s meditation library at the 24-week follow-up. Distribution of low, medium and high engagement in each respective program component is depicted in Table 2. Participants’ engagement scores were categorized as low, medium, and high, with some categories including more modules or more days of app use, as the response options could not be divided into three equal categories (0–10 for number of completed modules and 0–7 for days of app usage per week). Which category included a larger number of modules/days was decided upon examination of the data and after discussion within the research team. Detailed analyses of adherence and engagement with the nuumi program’s features in the first 8 weeks after program initiation have been reports elsewhere [48].

**Table 2:** Nuumi program component utilization at the 12- and 24-week follow-ups

Nuumi program component	Engagement at t_3_	Engagement at t_4_
App usage (days per week; M (SD))	2.81 (2.62)	0.94 (1.57)
Low (0–1 days; n (%))	27 (42.7)	41 (75.9)
Medium (2–4 days; n (%))	18 (28.6)	11 (20.4)
High (5–7 days; n (%))	18 (28.6)	2 (3.7)
Modules completed (M, SD)	4.63 (2.81)	4.89 (3.06)
Low (0–3 modules; n (%))	28 (44.4)	24 (44.4)
Medium (4–7 modules; n (%))	23 (30.1)	14 (26.0)
High (8–10 modules; n (%))	12 (19.0)	16 (29.6)
Meditation library usage (minutes; M (SD))	34.85 (66.9)	46.98 (112.79)
Low (0–10 min; n (%))	27 (45.0)	26 (51.0)
Medium (11–60 min; n (%))	24 (40.0)	18 (35.3)
High (61–400 min; n (%))	9 (15.0)	7 (13.7)

#### Nuumi EC use and dependence

At 12 weeks, 60.3% (38/63) of participants who filled out the survey reported nuumi EC use in the past 7 days; these individuals reported they used the nuumi EC on an average of 5 (SD = 2.42) days per week. At 24 weeks, 27.8% (15/54) of individuals who filled out the survey reported nuumi EC use in the past 7 days, and reported to have used the nuumi EC on an average of 4.47 (SD = 2.36) days per week. EC dependence of the individuals who reported using the nuumi EC was on average 7.37 (SD = 3.89) at 12 weeks, and on average 4.40 (SD = 3.58) at 24 weeks. At 12 weeks, nuumi EC dependence of 7.9% (n = 3) was high, dependence of 31.6% (n = 12) was medium, dependence of 36.8% (n = 14) was low, and 23.6% (n = 9) were not dependent. At 24 weeks, nuumi EC dependence of 0% was high, dependence of 20.0% (n = 3) was medium, dependence of 40.0% (n = 6) was low, and 40.0% (n = 6) were not dependent.

### Smoking abstinence

**Fig. 5:**
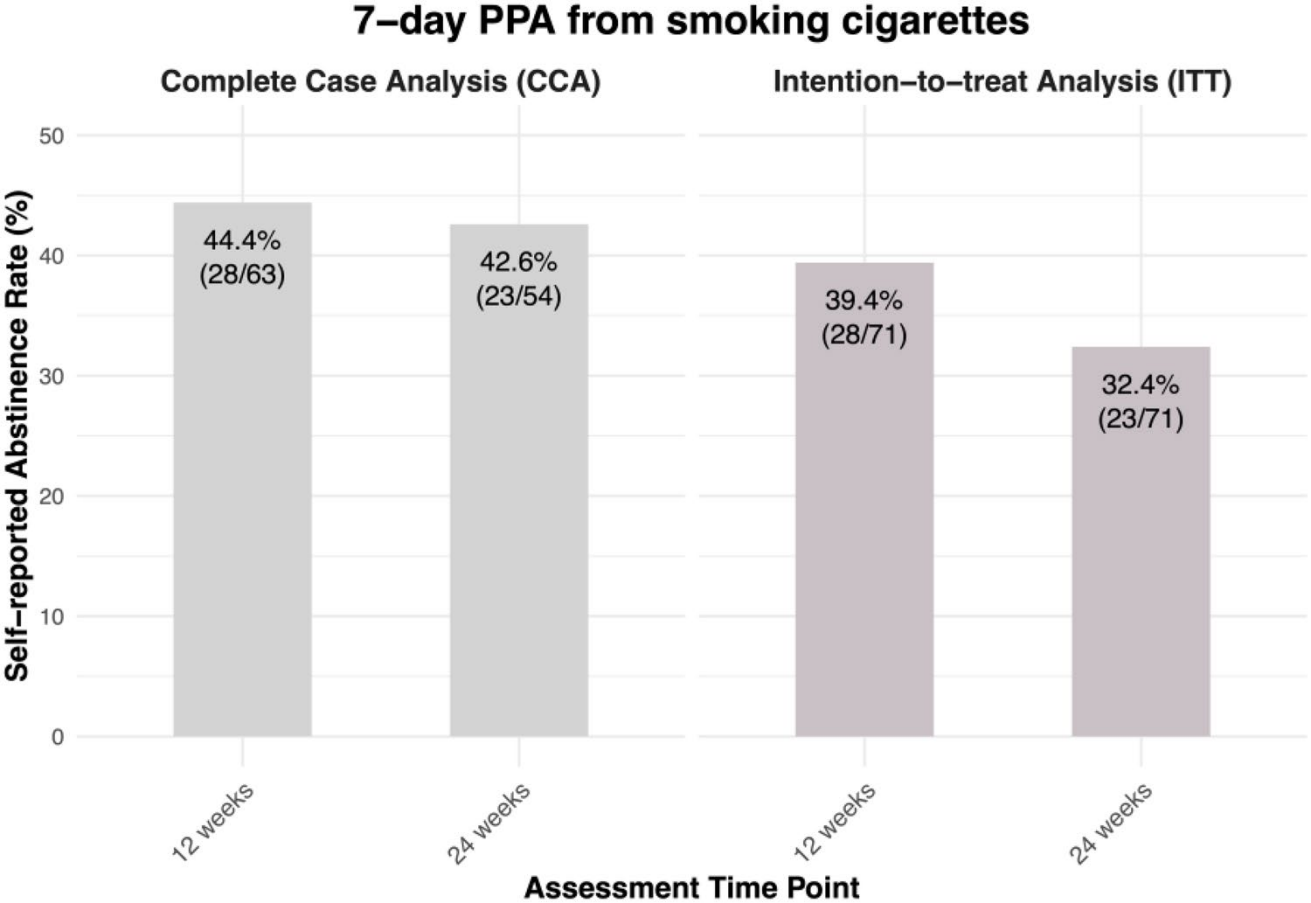
7-day PPA from smoking cigarettes at 12 weeks and 24 weeks post-baseline, based on Complete Case Analysis (CCA) and Intention-to-treat Analysis (ITT)

Self-reported 7-day smoking abstinence data were available for 88.7% (63/71) of participants at 12 weeks post-baseline, and for 76.1% (54/71) of participants at 24 weeks post-baseline. At 12 weeks post-baseline, 44.4% (28/63) of participants who completed the 12-week survey reported that they had not smoked cigarettes in the past 7 days (39.4% of all enrolled participants (ITT)), see Fig. 5. At 24 weeks post-baseline, 42.6% (23/54) of participants who completed the 24-week survey self-reported that they did not smoke cigarettes in the past 7 days (32.4% of all enrolled participants (ITT)), see Fig. 5. More information on 7-day PPA from smoking cigarettes is reported in Table A 2 of Appendix A.

Participants who reported smoking abstinence at 12 weeks had reported significantly fewer CPD at baseline (M = 15.18) relative to those who reported non-abstinence (M = 20.03; *p* < 0.001; see Table 3), and participants who reported smoking abstinence at 24 weeks had reported significantly fewer CPD at baseline (M = 14.17) relative to those who reported non-abstinence (M = 19.42; *p* < 0.01; see Table 3). Therefore, we controlled for variable baseline CPD in the subsequent GLMMs exploring the relationships of changes in urges to smoke, and psychophysiological health outcomes and smoking status.

**Table 3:** Baseline characteristics by smoking status at 12- and 24-weeks follow-ups

Characteristics	12 weeks post-baseline			24 weeks post-baseline		
	Abstinent individuals	Non-abstinent individuals	*p*	Abstinent individuals	Non-abstinent individuals	*p*
Age, M (SD)	36.82 (14.55)	41.43 (10.97)	0.171	37.17 (15.19)	40.71 (11.33)	0.354
Gender, n (%)			0.929			0.106
Male	10 (35.71)	11 (31.43)		10 (43.48)	6 (19.35)	
Female	18 (64.29)	24 (68.57)		18 (56.52)	25 (80.65)	
Diverse	0 (0)	0 (0)		0 (0)	0 (0)	
Higher education, n (%)			0.200			0.182
College degree	15 (53.57)	12 (34.29)		14 (60.87)	12 (38.71)	
No college degree	13 (46.43)	23 (65.71)		9 (39.13)	19 (61.29)	
CPD, M (SD)	14.18 (5.94)	20.03 (5.77)	< 0.001***	14.17 (6.71)	19.42 (6.05)	0.005**

Independent-group t-tests were conducted for continuous variables (age, CPD), and chi-square tests were conducted for categorical variables (gender, higher education)

*p < 0.05; **p < 0.01; ***p < 0.001

Table 4 shows the distribution of nuumi EC and cigarette use among the sample. At 12 weeks, 17.5% (11/63) of participants reported abstinence from both cigarettes and the nuumi EC, and 24 weeks, 31.5% (17/54) of participants reported abstinence from both cigarettes and the nuumi EC. Per ITT, these reflect 15.5% (11/71) at 12 weeks, and 23.9% (17/71) at 24 weeks of the total sample.

**Table 4:** Cigarette and nuumi EC use at the 12- and 24-week follow-ups

		12 weeks n = 63		24 weeks n = 54	
		nuumi EC use		nuumi EC use	
		Yes	No	Yes	No
Cigarettes	Yes	21 (33.3%)	14 (22.2%)	9 (16.7%)	22 (40.7%)
	No	17 (27.0%)	11 (17.5%)	6 (11.1%)	17 (31.5%)

### Secondary outcomes

#### 30-days PPA and repeated PPA

**Fig. 6:**
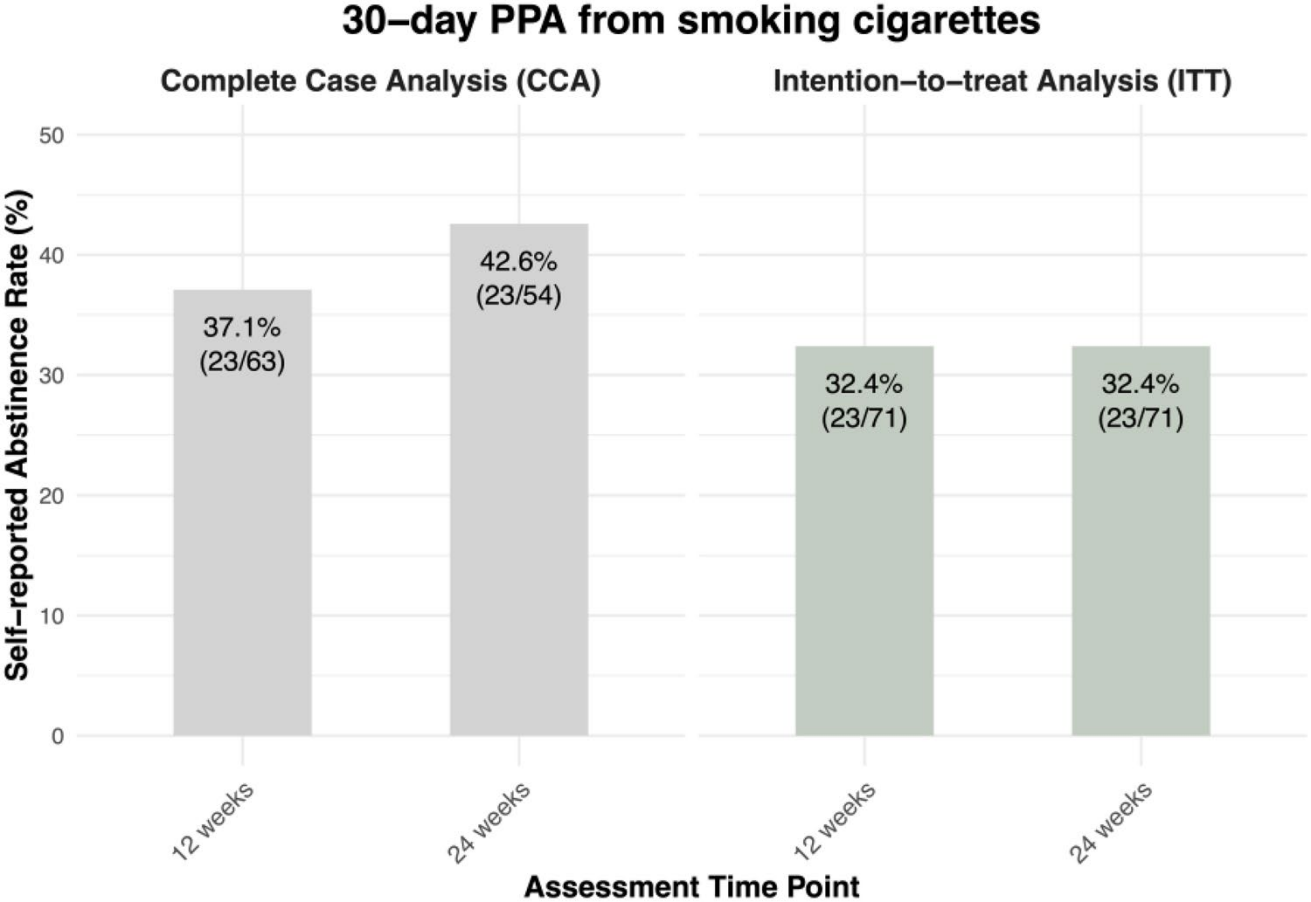
30-day PPA from smoking cigarettes at 12 and 24 weeks post-baseline, based on Complete Case Analysis (CCA) and Intention-to-treat Analysis (ITT)

At 12 weeks, 37.10% (23/63), and at 24 weeks 42.59% (23/54) of participants reported not having smoked any cigarettes in the past 30 days, see Fig. 6. Per ITT, at both 12 and 24 weeks, 32.39% (23/71) of participants reported not having smoked any cigarettes in the past 30 days, see Fig. 6. Repeated PPA from smoking cigarettes per ITT was 22.53% at both 12 and 24 weeks. Specifically, at 12 weeks, 16 enrolled participants reported 7-day PPA at the 4-, 8-, and 12-week follow-ups. Similarly, at 24 weeks, 16 participants reported 7-day PPA at the 4-, 8-, 12-, and 24-week follow-ups. More descriptive information on secondary outcomes is reported in Table A 3 of Appendix A.

#### CPD and urges to smoke

Participants who did not report 7-day PPA from smoking cigarettes at 12 weeks and completed the 12-week survey (55.6%, 35/63), reported an average of 12.74 (SD = 7.69) CPD, and at 24 weeks, participants who reported continued smoking (57.41%), reported an average of 14.77 (SD = 7.36) CPD. Compared to baseline, non-abstinent participants smoked significantly fewer CPD at both 12 weeks (*t*(34) = 6.12, *p* < 0.001, Cohen’s d = 1.07), and at 24 weeks (*t*(30) = 6.38, *p* < 0.001, Cohen’s d = 0.69). Compared to baseline, frequency of urges to smoke assessed by the VRS had decreased significantly at both the 12- and 24-week follow-ups, and intensity of urges to smoke had decreased significantly from baseline to the 12-week follow-up (see Table 5).

**Table 5:** Changes in VRS from the 12- and 24-week follow-ups

VRS	Baseline (M, SD)	12 weeks (M, SD)	*df*	*t*	*p*	Cohen’s *d*
Urge frequency	4.03 (0.62)	3.11 (1.32)	62	5.92	< 0.001***	0.89
Urge intensity	3.78 (0.82)	3.33 (1.10)	53	2.52	0.015*	0.45

	Baseline (M, SD)	24 weeks (M, SD)	*df*	*t*	*p*	Cohen’s *d*
Urge frequency	3.98 (0.60)	3.07 (1.49)	53	5.19	< 0.001***	0.80
Urge intensity	3.66 (0.76)	3.54 (0.90)	40	0.76	0.452	0.11

VRS = Urges to Smoke Scale; available *n* at 12 weeks = 63, at 24 weeks = 54

*p < 0.05, **p < 0.01, ***p < 0.001;

Bivariate analyses by smoking status revealed significant decreases in urge frequency and urge intensity for individuals who reported abstinence at 12 and at 24 weeks, while no changes in urge frequency and urge intensity were observed for individuals who reported non-abstinence at the two time points (see Table 6).

**Table 6:** Bivariate analyses of VRS by smoking status at the 12- and 24-week follow-ups

VRS	Abstinent individuals at t_3_					Non-abstinent individuals at t_3_				
	Score at t_**0**_ M (SD)	Score at t_3_ M (SD)	*p*	*t*(df)	*d*	Score at t_0_ M (SD)	Score at t_3_ M (SD)	*p*	*t*(df)	*d*
Urge frequency	3.75 (0.52)	2.11 (1.07)	< 0.001***	7.70 (27)	1.96	4.26 (0.61)	3.91 (0.89)	0.050	2.03 (34)	-
Urge intensity	3.75 (0.84)	2.53 (0.84)	< 0.001***	5.55 (18)	1.27	3.77 (0.81)	3.77 (0.97)	1	0 (34)	-

	Abstinent individuals at t_4_					Non-abstinent individuals at t_4_				
	Score at t_0_ M (SD)	Score at t_4_ M (SD)	*p*	*t*(df)	*d*	Score at t_0_ M (SD)	Score at t_4_ M (SD)	*p*	*t*(df)	*d*
Urge frequency	3.70 (0.47)	1.65 (0.88)	< 0.001***	10.04 (22)	2.88	4.19 (0.60)	4.13 (0.81)	0.625	0.40 (30)	–
Urge intensity	3.65 (0.78)	2.50 (0.71)	< 0.001***	6.09 (9)	1.93	3.61 (0.76)	3.87 (0.67)	0.088	−1.76 (30)	–

t_0_ = Baseline; t_3_ = 12-week follow-up; t_4_ = 24-week follow-up; VRS = urges to smoke; *d* = Cohen’s d; available *n* at t_3_ = 63, at t_4_ = 54

*p < 0.05, **p < 0.01, ***p < 0.001

We employed logistic regression models to analyze whether urges to smoke are associated with smoking cessation at the 12- and 24-week follow-ups (see Table 7). The results revealed that, when controlling for CPD and VRS scores at baseline, lower scores of urge frequency were significantly associated with greater odds of smoking cessation at 12 weeks (*p* = 0.003) and 24 weeks (*p* = 0.013), and lower scores of urge intensity were also significantly associated with greater odds of smoking cessation at 12 weeks (*p* = 0.013) and 24 weeks (*p* = 0.030).

**Table 7:** Relationship between VRS and smoking status at 12 and 24 weeks post-baseline

VRS	12 weeks			24 weeks		
	ß	Adjusted* p*	OR (95% CI)	ß	Adjusted* p*	OR (95% CI)
Urge frequency	−1.72	0.003**	0.18 (0.06–0.40)	−3.35	0.013*	0.03 (0.00–0.18)
CPD at t_0_	−0.10	0.254	0.91 (0.78–1.03)	0.09	0.493	1.10 (0.87–1.46)
Urge frequency at t_0_	−0.92	0.338	0.40 (0.07–1.66)	−0.54	0.723	0.58 (0.02–9.16)
Urge intensity	−1.72	0.013*	0.18 (0.05–0.47)	−4.59	0.030*	0.01 (0.00–0.12)
CPD at t_0_	−0.12	0.151	0.89 (0.78–1.01)	0.12	0.438	1.12 (0.88–1.56)
Urge intensity at t_0_	0.48	0.438	1.61 (0.63–4.92)	2.09	0.165	8.06 (1.17–211.89)

VRS = urges to smoke; *p*-values were adjusted for multiple comparisons using the BH method to control the FDR

*p < 0.05, **p < 0.01, ***p < 0.001

#### Psychophysiological health outcomes

Compared to baseline, a significant increase was observed at 12 weeks for SEQ-12 (*p* = 0.003), and SF12 PH scores (*p* = 0.005) at sample level (see Table 8). No significant changes were observed for PSS-10, FFA, L-1, and SF12 MH scores from baseline to 12 weeks (see Table 8). From baseline to 24 weeks, PSS-10 scores significantly decreased (*p* = 0.011). FAA scores, SEQ-12 scores, L-1 scores, SF12 PH scores, and SF12 MH scores significantly increased (all *p*s < 0.026, see Table 8).

**Table 8:** Changes in psychophysiological health outcomes on sample level from baseline to the 12- and 24-week follow-up

Outcome	Baseline (M, SD)	12 weeks (M, SD)	*df*	*t*	*p*	Cohen’s *d*
PSS-10	18.83 (5.01)	17.57 (5.54)	62	1.76	0.082	–
FFA	2.57 (0.38)	2.63 (0.34)	62	−1.40	0.167	–
SEQ-12	32.73 (32.73)	37.52 (10.95)	62	−3.06	0.003**	0.49
L-1	5.92 (1.62)	6.44 (1.86)	62	−1.87	0.066	–
SF12 PH	16.11 (2.24)	16.90 (2.08)	62	−2.92	0.005**	0.37
SF12 MH	18.87 (3.23)	19.52 (3.89)	62	−1.29	0.201	–

	Baseline (M, SD)	24 weeks (M, SD)	*df*	*t*	*p*	Cohen’s *d*
PSS-10	18.80 (5.17)	16.69 (5.94)	53	2.63	0.011*	0.38
FFA	2.56 (0.38)	2.71 (0.37)	53	−2.93	0.005**	0.38
SEQ-12	32.91 (8.80)	37.87 (13.41)	53	−2.90	0.026*	0.44
L-1	5.87 (1.65)	6.98 (2.11)	53	−3.23	0.002**	0.59
SF12 PH	16.13 (2.11)	16.89 (2.23)	53	−2.67	0.010*	0.35
SF12 MH	18.70 (3.36)	20.83 (3.77)	53	−4.06	< 0.001***	0.60

PSS-10 = Perceived Stress Scale, FFA = Freiburg Mindfulness Inventory, SEQ-12 = Smoking Self-Efficacy Questionnaire, L-1 = Short Scale Life Satisfaction-1, SF12 PH = Short Form Health Survey Physical Health, SF12 MH = Short Form Health Survey Mental Health; available *n* at t_3_ = 63, at t_4_ = 54

*p < 0.05, **p < 0.01, ***p < 0.001

Changes in psychophysiological health variables by smoking status at the 12- and 24-week follow-ups are depicted in Table 9. Compared to baseline, significant decreases in PSS-10 scores, and significant increases in FFA, SEQ-12, L-1, SF12 PH, and SF12 MH scores were observed for abstinent individuals at both 12 and 24 weeks (all *p*s < 0.005) For non-abstinent individuals, none of these scores changed from baseline to 12 weeks, and from baseline to 24 weeks, the only significant change observed was a decrease in SEQ-12 scores (*p* = 0.035).

**Table 9:** Bivariate analyses of changes in psychophysiological health outcomes by smoking status at the 12- and 24-week follow-ups

Outcome	Abstinent individuals at 12 weeks					Non-abstinent individuals at 12 weeks				
	t_0_ M (SD)	t_3_ M (SD)	*p*	*t (df)*	*d*	t_0_ M (SD)	t_3_ M (SD)	*p*	*t (df)*	*d*
PSS-10	19.25 (4.81)	14.89 (4.22)	< 0.001***	5.10 (27)	0.97	18.49 (5.22)	19.71 (5.58)	0.176	−1.38 (34)	–
FFA	2.45 (0.32)	2.70 (0.33)	< 0.001***	−3.85 (27)	0.75	2.66 (0.40)	2.57 (0.34)	0.083	1.79 (34)	–
SEQ-12	33.83 (8.93)	44.5 (9.74)	< 0.001***	−4.71 (27)	1.14	31.83 (8.03)	31.94 (8.44)	0.951	−0.06 (34)	–
L-1	5.57 (1.57)	7.14 (1.43)	< 0.001***	−3.75 (27)	1.05	6.20 (1.62)	5.89 (1.98)	0.324	1 (34)	–
SF12 PH	16.21 (2.08)	17.42 (1.45)	0.005**	−3.06 (27)	0.68	16.03 (2.39)	16.49 (2.42)	0.222	−1.24 (34)	–
SF12 MH	18.79 (3.37)	20.82 (2.76)	0.011*	−2.75 (27)	0.66	18.95 (3.15)	18.49 (4.37)	0.475	0.72 (34)	–

	Abstinent individuals at 24 weeks					Non-abstinent individuals at 24 weeks				
	t_0_ M (SD)	t_4_ M (SD)	*p*	*t (df)*	*d*	t_0_ M (SD)	t_4_ M (SD)	*p*	*t (df)*	*d*
PSS-10	18.83 (4.98)	13.38 (4.97)	< 0.001***	4.45 (22)	1.01	18.77 (5.39)	18.81 (5.76)	0.974	−0.03 (30)	–
FFA	2.49 (0.31)	2.78 (0.35)	< 0.001***	−3.55 (22)	0.85	2.61 (0.42)	2.65 (0.38)	0.499	−0.68 (30)	–
SEQ-12	32.30 (7.81)	50.17 (9.77)	< 0.001***	−7.63 (22)	2.02	33.35 (9.57)	28.74 (6.76)	0.035*	2.21 (30)	0.56
L-1	5.61 (1.73)	7.83 (1.40)	< 0.001***	−4.89 (22)	1.41	6.06 (1.59)	6.35 (2.33)	0.521	−0.65 (30)	–
SF12 PH	16.22 (2.26)	17.74 (1.36)	0.002**	−3.46 (22)	0.82	16.06 (2.05)	16.26 (2.54)	0.579	−0.56 (30)	–
SF12 MH	18.91 (3.68)	22.57 (2.48)	< 0.001***	−4.47 (22)	1.16	18.55 (3.15)	19.55 (4.06)	0.118	−1.61 (30)	–

t_0_ = Baseline; t_3_ = 12-week follow-up; t_4_ = 24-week follow-up; PSS-10 = Perceived Stress Scale, FFA = Freiburg Mindfulness Inventory, SEQ-12 = Smoking Self-Efficacy Questionnaire, L-1 = Short Scale Life Satisfaction-1, SF12 PH = Short Form Health Survey Physical Health, SF12 MH = Short Form Health Survey Mental Health; *d* = Cohen’s d; available *n* at t_3_ = 63, at t_4_ = 5

*p < 0.05, **p < 0.01, ***p < 0.001

We also assessed whether psychophysiological health outcomes were associated with smoking cessation at the 12- and 24-week follow-ups by applying logistic regression models controlling for CPD at baseline and the baseline scores associated with each outcome variable (see Table 10). Results revealed that lower PSS-10 scores were significantly associated with greater odds of smoking abstinence at 12 weeks (*p* = 0.020) and 24 weeks (*p* = 0.038). Higher FFA scores were significantly associated with greater odds of smoking cessation at 12 weeks (*p* = 0.039), but not at 24 weeks. Significant associations with greater odds of smoking cessation were detected for SEQ-12 scores at 12 weeks (*p* = 0.006) and 24 weeks (*p* = 0.006). Higher L-1 scores were also significantly associated with greater odds of smoking cessation at 12 weeks (*p* = 0.011), but not at 24 weeks. For SF12 MH scores, significant associations with greater smoking cessation odds were detected at 24 weeks (*p* = 0.039), but not at 12 weeks. No significant association between SF12 PH scores and smoking cessation was found.

**Table 10:** Relationship between psychophysiological health outcomes and smoking cessation at the 12- and 24-week follow-ups

Outcome	12-weeks			24-weeks		
	ß	Adjusted* p*	OR (95% CI)	ß	Adjusted* p*	OR (95% CI)
PSS-10	−0.27	0.020*	0.76 (0.63–0.89)	−0.21	0.038*	0.81 (0.69–0.94)
CPD at t_0_	−0.14	0.039*	0.87 (0.77–0.96)	−0.10	0.093	0.90 (0.81–0.99)
PSS-10 at t_0_	0.13	0.139	1.14 (0.99–1.33)	0.08	0.434	1.08 (0.93–1.26)
FFA	3.74	0.039*	41.90 (3.35–1098.01)	1.88	0.147	6.56 (0.83–67.54)
CPD at t_0_	−0.16	0.023*	0.85 (0.75–0.94)	−0.11	0.061	0.90 (0.81–0.98)
FFA at t_0_	−3.70	0.023*	0.02 (0.00–0.22)	−1.82	0.148	0.16 (0.02–1.17)
SEQ-12	0.15	0.006**	1.16 (1.08–1.27)	0.23	0.006**	1.26 (1.14–1.47)
CPD at t_0_	−0.19	0.023*	0.83 (0.71–0.93)	−0.05	0.582	0.95 (0.80–1.11)
SEQ-12 at t_0_	0.01	0.856	1.01 (0.93–1.11)	−0.04	0.584	0.96 (0.85–1.08)
L-1	0.56	0.039*	1.02 (1.18–2.86)	0.37	0.088	1.44 (1.04–2.14)
CPD at t_0_	−0.16	0.023*	0.85 (0.75–0.94)	−0.11	0.061	0.89 (0.80–0.98)
L-1 at t_0_	−0.48	0.078	0.62 (0.37–0.93)	−0.26	0.270	0.77 (0.51–1.11)
SF12 PH	0.19	0.417	1.21 (0.86–1.79)	0.50	0.064	1.65 (1.11–2.76)
CPD at t_0_	−0.15	0.003**	0.86 (0.77–0.94)	−0.12	0.056	0.89 (0.80–0.98)
SF12 PH at t_0_	−0.05	0.735	0.95 (0.70–1.29)	−0.24	0.286	0.79 (0.54–1.13)
SF12 MH	0.21	0.056	1.24 (1.00–1.35)	0.33	0.039*	1.39 (1.11–1.97)
CPD at t_0_	−0.16	0.020*	0.85 (0.77–0.94)	−0.12	0.049*	0.88 (0.80–0.97)
SF12 MH at t_0_	−0.07	0.549	0.93 (0.77–1.12)	−0.07	0.548	0.93 (0.76–1.13)

t_0_ = Baseline; t_3_ = 12-week follow-up; t_4_ = 24-week follow-up; p-values were adjusted for multiple comparisons using the BH method to control the FDR

*p < 0.05, **p < 0.01, ***p < 0.001

#### Acceptability

Using z-standardization, a mean acceptability score was calculated, and acceptability scores were divided in low (0–1.66), medium (1.67–3.32), and high (3.33–5). On average, the acceptability of the nuumi program was rated as 3.61 (SD = 0.87) at 12 weeks, where 1.6% (1/63) reported low, 31.7% (20/63) medium, and 66.7% (42/63) high acceptability. At 24 weeks, participants rated the program at an acceptability score of 3.54 (SD = 0.97), where 3.7% reported low (2/54), 42.6% (23/54) medium, and 53.7% (29/54) reported high acceptability. Detailed ratings of each component of the acceptability score are included in Table A 3 of Appendix A. The usability of the app, assessed by the SUS at 24 weeks, was rated at 79.68 (SD = 13.15).

#### Other smoking-behavior related variables

At 12 weeks, 9 participants (14.3%) reported use of other ECs, and at 24 weeks, 6 participants (11.1%) reported use of other ECs. None of the participants reported participation in any other smoking cessation program, neither at 12 weeks, nor at 24 weeks. At 12 weeks, no participants reported using NRT, while at 24 weeks, one participant reported current use of NRT. This participant reported use of NRT for 2 days within the past 7 days. At 12 weeks, 10 participants (15.9%) indicated they used alternative tobacco products in the past 7 days; these individuals reported they used alternative tobacco products for an average of 4 (SD = 2.67) days. At 24 weeks, 7 participants (13.0%) reported having past 7-day use of alternative tobacco products; the average number of days on which alternative tobacco products had reportedly been used in the past 7 days was 5.20 (SD = 2.36) days. More descriptive information on other outcomes is provided in Table A 4 of Appendix A.

## Discussion

This study evaluated the initial smoking cessation rates, acceptability, and psychophysiological health outcomes of nuumi, a novel intervention combining app-based behavioral therapy and an EC in a real-world setting. Results show that nuumi could potentially support adults who smoke in quitting smoking and may be associated with improvements in smoking cessation-related psychophysiological health outcomes. Response rates of 88.7% at the 12-week follow-up, and 76.1% at the 24-week follow-up are comparable with median retention rates of 80% typically observed in RCTs of behavioral smoking cessation interventions [62].

### Smoking cessation

Our results are in line with those of previous studies suggesting that digital behavioral therapy [39] and EC use [15] may encourage smoking cessation. At 12 weeks post-baseline, 39.4%, and at 24 weeks post-baseline, 32.4% of participants self-reported 7-day PPA from smoking cigarettes. Thirty days PPA was reported by 32.4% of participants at both follow-ups. Taking into account that the unique features of nuumi, and different outcome measurements in smoking cessation trials limit comparability of results, outcomes of the nuumi program compare favorably to other interventions. For example, a recent randomized controlled trial found that providing individuals who smoke with a nicotine EC plus behavioral counselling resulted in a biochemically verified 7-day PPA from smoking cigarettes of around 22% 12 weeks after baseline [63], and a biochemically verified 7-day PPA from smoking cigarettes of 23% has been reported in another EC study [64]. Moreover, 24-weeks abstinence rates in studies investigating mobile interventions for smoking cessation have been shown to lie within the range of 4% to 22%, depending on the abstinence measurement and intervention [39]. Hence, the self-reported 7-day PPA from smoking cigarettes rates reported in the present study suggest that the nuumi intervention may hold some promise to support adults who want to quit smoking in achieving abstinence; however, a causal relationship cannot be established due to the single-arm design of our trial.

### CPD and urges to smoke

We further found that study completers who did not achieve abstinence reduced their number of CPD significantly at both the 12-week and 24-week follow-ups. Reducing the number of cigarettes has been shown to increase the likelihood of future smoking cessation, as individuals who decrease their smoking behavior are more likely to attempt and succeed in quitting later [65]. Although some evidence shows that a substantial reduction in the number of CPD may be linked to lower risk of cardiovascular disease and respiratory symptoms, and may be associated with a lower risk of lung cancer [66], even low rates of smoking are associated with increased rates of morbidity and mortality compared to not smoking [67, 68]. We further observed that urges to smoke decreased significantly over the study period among participants who self-reported successful cessation, and when controlling for baseline CPD and baseline levels of urges to smoke, lower scores of both urge frequency and urge intensity were associated with greater odds of smoking cessation at the 12-week and 24-week follow-ups. Urges to smoke are an indicator of cigarette dependence, and a strong predictor of smoking cessation [69]. A possible explanation for the decrease in urges to smoke in abstinent individuals is that having access to the nuumi EC may have potentially reduced smoking urges as ECs have been previously suggested to do so [70] even when not containing nicotine [71], perhaps due to mimicking the hand-to-mouth action and inhalation process associated with cigarette smoking [72]. Also, using the behavioral support components of the program may have played a role in the observed reduction of self-reported cravings among participants as previous research has suggested that CBT- and MII-based interventions may potentially enhance affect regulation, which may in turn help manage affective states that trigger smoking urges [28]. As the present study was a non-randomized pilot study, the results cannot be conclusively attributed to the intervention. It is possible that the observed reduction in smoking urges could be due to latent variables unrelated to the intervention.

### Psychophysiological health outcomes

Participants reported significant improvements in perceived stress, mindfulness, smoking self-efficacy, life satisfaction, subjective physical health and mental health from baseline to the end of the 6 months study period. Importantly, bivariate analyses revealed that these changes were almost exclusively present in individuals who reported to have quit smoking, which is in line with evidence showing that smoking cessation is associated with improvements in several psychophysiological health outcomes [3, 4].

When controlling for baseline scores of the respective variable and baseline CPD, and adjusting p-values for multiple comparisons, lower perceived stress, and higher smoking self-efficacy were found to be significantly associated with greater smoking cessation odds both 12 and 24 weeks after nuumi program initiation. Evidence suggests that stress is often a precipitant of relapses to smoking [73], and CBT-based and mindfulness-informed interventions that target regulation of stress and affective states [23, 74] can increase the likelihood of successful cessation [38]. The relationship between stress and smoking cessation observed among our participants may reflect the bidirectional relationship between perceived stress and smoking that has been previously described [75–77]. On the one hand, learning to cope with stress and reducing stress levels may facilitate smoking cessation [76]. On the other hand, successful cessation may lead to reduced levels of perceived stress [75, 77]. Nicotine dependence itself may be a source of stress; supplying oneself with sufficient nicotine and experiencing withdrawal symptoms when nicotine levels drop creates a cycle of symptoms like stress and anxiety, followed by relief once nicotine is obtained [78, 79]. By quitting smoking, individuals may break this cycle, leading to an overall reduction in perceived stress over time.

Our findings further align with a substantial body of research showing that self-efficacy is a strong predictor of smoking cessation success, and that enhancing self-efficacy through interventions like CBT may improve the chances of quitting [80, 81]. Features of the nuumi program may have served as useful tools for some participants, helping them refrain from smoking and facilitating a sense of mastery which in turn may have increased feelings of self-efficacy [82, 83]. Enhancing self-efficacy may have created a positive feedback loop, reinforcing the cessation process.

Further, subjective mental health was significantly associated with increased odds of successful smoking cessation by the end of the study period. This finding is in line with some previous research showing smoking cessation to be associated with improvements in mental health outcomes like anxiety and depression [3]. One possible explanation for the link between smoking cessation and improved mental health is the disruption of the cycle of smoking and withdrawal symptoms like anxiety and stress mentioned previously [3, 79]. The tobacco dependence cycle may create a baseline of heightened anxiety and stress for individuals who smoke, as they are continually managing the physical and psychological effects of nicotine depletion. By quitting, though still speculative, individuals may break free from this cycle, which may lead to an overall improvement in mental well-being.

We observed an initial association between mindfulness and smoking cessation at 12 weeks after baseline, although the confidence interval was wide. By 24 weeks, however, this association was no longer present. Some evidence suggests that mindfulness reduces overall stress [84], and helps weaken the link between cravings and smoking [26, 27], which might play a role in the initial weeks of cessation. Literature on the long-term effects of mindfulness interventions for smoking cessation remains inconsistent [29–32]. Mindfulness has been hypothesized to be developed through consistent practice [85, 86]. Participants’ limited engagement with the nuumi intervention in our study may not have been sufficient for participants to cultivate the mindfulness skills necessary to manage cravings and prevent relapse over the long term.

Finally, subjective physical health was not significantly associated with smoking cessation when controlling for baseline CPD and baseline physical health scores. This lack of association could be due to the relatively short time frame of the study; improvements in physical health may not have fully manifested or affected participants' ability to remain abstinent in the longer term. Additionally, it's important to consider that the SF12 PH scale may not capture all the relevant dimensions of health that are associated with smoking. While the SF12 is a reliable measure of general physical health [87], it does not specifically assess respiratory function or other smoking-related health markers, which might be more directly linked to smoking cessation. Future studies may use different measures like the Medical Research Counsil (mMRC) Dyspnea Scale which measures the level of breathlessness experienced during different activities and reflects short-term improvements in lung function and breathing [88].

As previously stated, the design of our study prevents us from pinpointing what nuumi factors, if any, may have affected the trajectory of psychophysiological health outcomes, and their respective relationship with smoking cessation, and we can only make assumption about directionalities of associations. Moreover, although our sample size estimation was based on similar studies and recommended standards for sample sizes in single-arm pilot studies [42], our small sample size may have limited our ability to detect some associations between smoking cessation and the described outcomes. A well-powered RCT is needed to explore pathways through which participation in the nuumi program is associated with psychophysiological health outcomes and smoking cessation. A larger sample size would also enable us to investigate the relationships between the measured variables in more complex regression models, providing insights into the factors that may potentially mediate or moderate these associations.

### Acceptability

Usability of the nuumi intervention was rated using the System Usability Scale, a widely used scale for the assessment of user-friendliness of digital systems. Ratings were high at 79.68, suggesting an above standard usability level compared to benchmarking scores for digital health apps [89]. More than 95% of participants reported medium to high acceptability ratings for the nuumi program at both 12 and 24 weeks, suggesting that nuumi presents an intervention that was perceived as helpful in not smoking cigarettes, increased participants’ confidence in quitting smoking, and was rated as highly satisfactory.

### Adherence and engagement

#### App and behavioral therapy use

Behavioral therapy engagement shows room for improvement; on average, participants had finished around 50% of the behavioral therapy modules by the end of the study period, and meditation library usage was low. Evidence shows that adherence is generally low in digital smoking cessation interventions, and such programs may therefore fail to achieve long-term cessation rates [90, 91]. Steps to increase engagement with the nuumi app may be warranted and could include more gamification elements like achievements, challenges or reward systems [92], or social support features such as community forums or peer-to-peer messaging [93]. Community features may have potential to improve engagement and likelihood of smoking abstinence [94, 95]. Additionally, peer interaction may support smoking cessation by providing users with a sense of autonomy, emotional, informational, and instrumental support, and by shaping perceived social norms that encourage quitting [96]. Providing content tailored to the individual characteristics of each user, such as personalized counseling messages or digital face-to-face coaching, could further enhance engagement [97, 98]. As part of the present trial, interviews were conducted to evaluate the intervention qualitatively, and their results may offer valuable insights for enhancing engagement with the intervention.

#### Electronic cigarette use

Twelve weeks after intervention initiation, 60.3% of participants reported having used the nuumi EC in the past 7 days, and 24 weeks after intervention start, 27.8% reported so. Of these individuals, 60.4% at 12 weeks, and 80.0% at 24 weeks reported low or no dependence on the EC. Participants were provided with EC pods for around 16 weeks of use and were recommended to follow a nicotine tapering schedule; thus, at the 12-week follow-up, use of the nuumi EC may be interpreted as an indicator of adherence as participants were instructed and therefore still expected to use their EC at this time, while this was no longer the case at the 24-week follow-up. Two patterns were reflected in the results; 11.1% of participants reported exclusive use of the nuumi EC in the past 7 days at the end of the study period, and 16.7% reported dual use of cigarettes and the nuumi EC at the 24-week follow-up. These participants may have completed the program at a slower pace or with interruptions and therefore still had pods available at 24 weeks after program initiation. Ongoing EC use may reflect the previously described problem of persistent EC use among individuals attempting to quit smoking using ECs [19]. The nuumi program incorporates support to cease EC use over time by asking participants to use pods with decreasing nicotine concentration while simultaneously adhering to a daily puff budget to prevent compensatory puffing. However, providing participants with a limited supply of pods per nicotine strength and implementing a puff budget may have led participants to decrease their nicotine intake prematurely, which may have placed them at risk for sudden rises in cravings, which in turn may have resulted in increased use of EC, cigarettes, or both. Previous research findings suggest that individuals interested in quitting smoking prefer refillable devices over closed-system devices [99]. Although some evidence suggests that tank systems are more frequently associated with quitting smoking than non-refillable devices [100], providing individuals with an open-system device allowing user-customized modifications including the adjustment of nicotine concentration may be associated with some risks, including exposure to substantially higher doses of nicotine, and use of liquids with harmful constituents non-compliant with regulation [101]. Furthermore, open EC systems that enable users to modify device power may facilitate compensation for lower nicotine concentrations, resulting in higher nicotine intake and enhanced nicotine reinforcement, which could increase dependence potential [102].

Previous literature has highlighted ECs as a potentially less harmful alternative to combustible cigarettes for individuals who quit smoking using ECs but are unable to abstain from both cigarettes and ECs [103]. Additionally, research findings suggest that switching from cigarette use to exclusive EC use may be linked to reduced odds of developing respiratory illnesses such as chronic obstructive pulmonary disease (COPD), chronic bronchitis, emphysema, and asthma [104]. However, no long-term data are available to conclusively determine health-related consequences of ongoing EC use; thus, EC-based smoking cessation efforts should address EC cessation as important part of their program. Similarly, no long-term data on health consequences are available to EC use or EC use in combination with cigarettes (dual use). A recent secondary analysis of a Cochrane systematic review shows no evidence that biomarkers of potential harm, including exhaled CO as well as some carcinogens and toxicants, are increased in individuals who smoke combustible cigarettes while also using an EC, and that dual use is associated with significant reductions in CO and various other toxic and cancer-causing chemicals compared to consuming cigarettes exclusively [105]. However, dual users may have higher overall nicotine intake and dependence than exclusive EC users [106, 107]. Dual users remain exposed to toxicants contained in cigarettes, and, as indicated previously, even low rates of smoking are associated with increased morbidity and mortality risks in comparison to not smoking [67]. Evidence regarding the role of dual use in smoking cessation is mixed. While some evidence suggests that dual use may be associated with an increased risk of relapse, with individuals returning to cigarette smoking [108], for some individuals, an intermediate period of dual use may increase the likelihood of smoking cessation, although only in the short-term [109].

Some adjustments may be made to the nuumi program to support participants ceasing EC use more intensively. For example, the program could integrate more educational content about the risks associated with dual use. Further, to improve smoking cessation, and in later stages of the program EC cessation rates, the nicotine tapering process could be personalized further, e.g. by giving participants a higher number of pods of each nicotine strength to choose from, and guide their use based on an app feature that allows them to report cravings or withdrawal symptoms in real-time and in turn adjusts the nicotine reduction pace or offers tailored motivational messages. More research is needed to investigate possibilities and barriers in nicotine tapering by using ECs.

### Limitations

The findings of the present pilot trial should be interpreted considering several limitations, which partly have previously been described in the trial protocol [40]. The lack of a control group prevents clear attribution of outcomes to the intervention itself and limits comparison with alternative treatments. Our eligibility criteria limit the generalizability of our findings. For example, our sample consisted exclusively of individuals who were highly motivated to quit smoking while levels of motivation to quit typically vary among the general population of individuals who smoke [110]. Additionally, the exclusion of individuals with severe psychiatric illnesses and substance use disorders resulted in a sample that was not representative of the general population of individuals who smoke, as the aforementioned groups constitute a substantial proportion of individuals who smoke [111]. However, conducting an online study would have made it difficult to recognize and address any mental health crises occurring during the cessation attempt, and the lack of in-person or phone contact with the participants throughout the study would have prevented us from ensuring the safety of the aforementioned vulnerable populations. Generalizability of our findings may also be limited given that the program was provided to participants at no cost, while there are costs associated with the program outside of the study. Receiving the program at no cost could have acted as a financial incentive. Some research suggests that incentives can improve smoking cessation outcomes and may continue to support sustained cessation even after the incentive is no longer issued [112]. Therefore, smoking cessation rates in our study might have been lower if participants had to pay for the program. Another limitation concerns the assessment of smoking abstinence rates solely through self-reported data, where misreporting may occur [113]. Future research should consider incorporating biochemical verification methods to enhance the validity and reliability of smoking abstinence assessments [113]. An additional limitation is the lack of in-lab testing of the EC; using an EC with an unknown nicotine delivery profile in this trial prevented us from knowing whether participants received enough nicotine to suppress cravings when using either of the nicotine concentrations provided as part of the gradual reduction approach chosen by the program manufacturer. Insufficient nicotine delivery at either or all of the reduction stages may have led to compensatory puffing [114], nicotine cravings and continued smoking [115]. More research is needed to examine this approach and identify if and to what degree a gradual reduction of nicotine has any effect on smoking cessation and if so, whether that effect helps or hinders successful cessation. Although participants could closely monitor their daily EC puffs and current nicotine concentration of the EC pods in the nuumi app, this data was not available to be analyzed within the framework of the present trial due to technical and resource constraints. The nuumi EC’s ability to track puffing patterns provides a valuable opportunity to gain insights into participants’ use patterns and may provide information on compensatory puffing behaviors during the smoking cessation process. Future studies should leverage these data to examine interactions of nicotine concentration, puff frequency, and puff duration over time. Additionally, should future research findings suggest that using nicotine tapering strategies could potentially provide a helpful cessation tool for individuals who smoke, analyzing puffing data could inform modifications of such strategies to optimize individuals’ transition from cigarettes to ECs and to complete cessation of both products, potentially improving intervention outcomes. In addition, we did not analyze for a possible specific effect of the chosen flavor on our outcomes or the maintenance of nuumi EC consumption at the end of treatment. However, both flavors available to participants were tobacco-flavored and did not differ substantially in their characteristics. Finally, as another methodological limitation, we conducted a series of logistic regressions to evaluate the association of changes in urges to smoke, and psychophysiological health outcomes with smoking cessation. While we corrected for multiple comparisons, we did not consider interactions between variables or fit a single comprehensive model including all predictors. This approach was necessitated by our limited sample size, which precluded the use of a more complex multivariate model due to potential issues with statistical power and model stability. As this analysis is part of the exploratory phase of research on this program, performing multiple logistic regressions helped identify which variables may be associated with smoking cessation outcomes to explore them in more detail in future research.

## Conclusions

Smoking continues to be a leading cause of preventable diseases, significantly burdening healthcare systems and society. Developing and implementing effective new smoking cessation interventions is crucial for improving both individual and public health outcomes. To the best of our knowledge, nuumi represents the first comprehensive smoking cessation program that integrates an EC with an app combining CBT- and mindfulness-based therapy content, thus offering a multi-level approach for individuals motivated to quit smoking. In this pilot investigation, we found that using the nuumi program may be associated with quit rates that compare favorably with smoking cessation outcomes reported in other peer-reviewed literature where smoking cessation interventions like ECs or digital behavioral therapy programs were investigated. Although this pilot study is limited in scope, its design adheres to established methodological standards for preliminary assessments of novel interventions. Our findings suggest that acceptability of the program was satisfactory, however, indices of program engagement suggest there is room for improvement. Moving forward, this study will guide nuumi program refinements and further evaluation through an RCT. Moreover, our findings help guide future development of smoking cessation programs including ECs and app-based therapies.

## Supplementary Information

Additional file 1

## Data Availability

The datasets used and/or analyzed during the current study are available from the corresponding author on reasonable request.

## Abbreviations

BH: Benjamini-Hochberg
CBT: Cognitive-behavioral therapy
CCA: Complete case analysis
COPD: Chronic obstructive pulmonary disease
CPD: Cigarettes per day
EC: Electronic cigarette
FDR: False discovery rate
ITT: Intention-to-treat
mHealth: Mobile health
MII: Mindfulness-informed intervention
mMRC: Medical Research Counsil
RCT: Randomized controlled trial
SD: Standard deviation
TAU: Treatment as usual
ZPP: Central Prevention Testing Center
