# Supplementary material for: Initial assessment of a novel smoking cessation program integrating app-based behavioral therapy and an electronic cigarette: results of a pilot study
Source: Addict Sci Clin Pract. 2025 Mar 27;20:31. doi: 10.1186/s13722-025-00559-w (PMC11948757; doi:10.1186/s13722-025-00559-w)
Supplement: Supplementary file 1 — Additional file 1 [file 13722_2025_559_MOESM1_ESM.docx]

**Appendix A – Additional Descriptive Results**

**Table A 1** Baseline participant characteristics (N = 71)

| Demographic characteristics | Statistics |
| --- | --- |
| Female, n (%) | 49 (69%) |
| Age, M (SD) | 39.78 (10.85) |
| Highest level of education, n (%) |  |
| General School Leaving Certificate | 7 (9.9) |
| Intermediate School Leaving Certificate | 20 (28.2) |
| University of Applied Sciences Entrance Qualification | 12 (16.9) |
| General University Entrance Qualification | 32 (45.1) |
| Professional degree, n (%) |  |
| No professional qualification | 9 (12.7) |
| Recognized professional training | 26 (36.6) |
| Master professional/technician or equivalent | 5 (7.0) |
| Bachelor’s degree or equivalent | 12 (16.9) |
| Master’s degree or equivalent | 16 (22.5) |
| Doctorate degree | 1 (1.4) |
| Other professional degree | 2 (2.8) |
| Employment status, n (%) |  |
| Student | 2 (2.8) |
| Employed full-time | 42 (59.2) |
| Employed part-time | 14 (19.7) |
| Employed on a marginal basis | 1 (1.4) |
| In professional training, retraining | 4 (5.6) |
| Disability pension | 2 (2.8) |
| Professional reintegration program | 1 (1.4) |
| Other | 5 (7.0) |
| Family status, n (%) |  |
| In a partnership, living together | 41 (57.8) |
| In a partnership, not living together | 11 (15.5) |
| Single | 19 (26.8) |
| Number of children, n (%) |  |
| 0 | 34 (47.9) |
| 1 | 17 (23.9) |
| 2 | 17 (23.9) |
| 3 | 1 (1.4) |
| 4 | 1 (1.4) |
| 5 | 1 (1.4) |
| Household size, M (SD) | 2.24 (1.0) |
| Household income, n (%) |  |
| < 1.000€ | 3 (4.2) |
| 1.000€ - 2.000€ | 11 (15.5) |
| 2.001€ - 3.000€ | 14 (19.7) |
| 3.001€ - 4.000€ | 16 (22.5) |
| 4.001€ - 5.000€ | 10 (14.1) |
| > 5.000€ | 17 (23.9) |
| Height in cm, M (SD) | 172.62 (10.34) |
| Weight in kg, M (SD) | 78.92 (22.37) |
| Cigarettes per day (CPD), M (SD) | 17.73 (6.45) |
| Years of smoking, M (SD) | 21.42 (10.91) |
| Nicotine dependence |  |
| M (SD) | 5.34 (2.29) |
| Low, n (%) | 10 (14.1) |
| Moderate, n (%) | 20 (28.2) |
| Strong, n (%) | 41 (57.8) |
| Quit attempts, n (%) |  |
| 0 | 5 (7.0) |
| 1-2 | 24 (33.8) |
| 3-4 | 25 (35.2) |
| 5-6 | 8 (11.3) |
| 7-8 | 2 (2.8) |
| 9-10 | 3 (4.2) |
| >10 | 4 (5.6) |
| Quit methods, n (%) |  |
| Willpower | 41 (57.8) |
| Allen Carr’s “Easy Way to Stop Smoking” | 24 (33.8) |
| App | 20 (28.2) |
| NRT without prescription | 20 (28.2) |
| Electronic cigarette with nicotine | 20 (28.2) |
| Social environment support | 15 (21.1) |
| Tobacco heater | 10 (14.1) |
| Electronic cigarette without nicotine | 8 (11.2) |
| Counselling by pharmacist | 7 (9.9) |
| Behavioral therapy | 7 (9.9) |
| Hypnotherapy | 7 (9.9) |
| Acupuncture | 7 (9.9) |
| Counselling by doctor | 5 (7.0) |
| Website | 5 (7.0) |
| Other books | 4 (5.6) |
| NRT with prescription | 4 (5.6) |
| Zybian/Bupropion | 3 (4.2) |
| Counselling by alternative practitioner | 3 (4.2) |
| Telephone counselling | 2 (2.8) |
| Champix/Varenicline | 1 (1.4) |
| VRS, M (SD) |  |
| Urge frequency | 4.07 (0.64) |
| Urge intensity | 3.73 (0.81) |
| PSS-10, M (SD) |  |
| M (SD) | 18.92 (5.01) |
| Low, n (%) | 10 (15.1) |
| Moderate, n (%) | 55 (77.5) |
| High, n (%) | 6 (8.5) |
| FFA (M, SD) | 2.57 (0.39) |
| SEQ-12, M (SD) | 33.42 (8.9) |
| SF12, M (SD) |  |
| SF12 PH | 16.07 (2.22) |
| SF12 MH | 18.80 (3.19) |
| L-1, M (SD) | 5.97 (1.67) |
| Meditation experience (yes), n (%) | 33 (46.5) |
| Open towards meditation (yes), n (%) | 64 (90.1) |

*Note.* VRS = Urges to Smoke Scale, PSS-10 = Perceived Stress Scale, FFA = Freiburg Mindfulness Inventory, SEQ-12 = Smoking Self-Efficacy Questionnaire, L-1 = Short Scale Life Satisfaction-1, SF12 PH = Short Form Health Survey Physical Health, SF12 MH = Short Form Health Survey Mental Health

| Primary outcome (7-day PPA) | 4 weeks post-baseline (t_1_),  n = 66 | 8 weeks post-baseline (t_2_),  n = 63 | 12 weeks post-baseline (t_3_), n = 62 | 24 weeks post-baseline (t_4_), n = 54 |
| --- | --- | --- | --- | --- |
| Complete cases analysis (CCA) | 36.4%  (24/66) | 41.9%  (26/62) | 44.4%  (28/63) | 42.6%  (23/54) |
| Intention-to-treat analysis (ITT) | 33.8%  (24/71) | 36.6%  (26/71) | 39.5%  (28/71) | 32.4%  (23/71) |

**Table A 2** Primary outcome at the 4-week, 8-week, 12-week, and 24-week follow-ups (N = 71)

**Table A 3** Descriptive statistics of secondary outcomes at the 4-week, 8-week, 12-week, and 24-week follow-ups (N = 71)

| Secondary outcomes | 4 weeks post-baseline (t_1_),  n = 66 | 8 weeks post-baseline (t_2_),  n = 62 | 12 weeks post-baseline (t_3_),  n = 63 | 24 weeks post-baseline (t_4_),  n = 54 |
| --- | --- | --- | --- | --- |
| Cigarettes per day (CPD), M (SD)^1^ | 14.05 (7.89) | 11.58 (7.79) | 12.74 (7.69) | 14.77 (7.36) |
| 30 days PPA | - | CCA: 30.7% (19/61)  ITT: 26.8% (19/71) | CCA: 36.5% (23/63)  ITT: 32.4% (23/71) | CCA: 42.6% (23/54)  ITT: 32.4% (23/71) |
| Repeated PPA | - | 23.9% (17/71) | 22.5 (16/71) | 22.5% (16/71) |
| Cigarette dependence, M (SD)^2^ | 4.90 (1.92) | 4.11 (2.47) | 4.63 (2.41) | 5.1 (2.34) |
| Low, n (%) | 11.9% (5/42) | 25.0% (9/36) | 17.1% (6/35) | 12.9% (4/31) |
| Moderate, n (%) | 47.6% (20/42) | 38.9% (14/36) | 37.1% (13/35) | 35.5% (11/31) |
| Strong, n (%) | 38.1% (16/42) | 36.1% (13/36) | 45.7% (16/35) | 51.6% (16/31) |
| Urges to smoke |  |  |  |  |
| Urge frequency, M (SD) | 3.42 (0.98) | 3.32 (1.10) | 3.11 (1.32) | 3.07 (1.49) |
| Urge intensity, M (SD) | 3.61 (1.00) | 3.36 (0.92) | 3.33 (1.10) | 3.54 (0.90) |
| Perceived stress |  |  |  |  |
| M (SD) | 18.91 (6.01) | 18.44 (5.83) | 17.57 (5.54) | 16.69 (5.94) |
| Low, n (%) | 10.6% (7/66) | 16.1% (10/62) | 23.8% (15/63) | 9.3% (5/54) |
| Moderate, n (%) | 71.2% (47/66) | 79.0% (49/62) | 71.4% (45/63) | 31.5% (17/54) |
| High, n (%) | 18.2% (12/66) | 4.8% (3/62) | 4.8% (3/63) | 59.3% (32/54) |
| Mindfulness (M, SD) | 2.49 (0.31) | 2.60 (0.42) | 2.63 (0.34) | 2.71 (0.37) |
| Smoking self-efficacy single-item, M (SD) | 6.98 (2.04) | 6.89 (2.12) | 6.29 (2.86) | 6.35 (3.26) |
| Smoking self-efficacy scale, M (SD) | 35.14 (8.73) | 37.92 (10.56) | 37.52 (10.95) | 37.87 (13.41) |
| Subjective health |  |  |  |  |
| SF12 PH, M (SD) | 16.24 (2.45) | 16.37 (2.48) | 16.90 (2.08) | 16.89 (2.23) |
| SF12 MH, M (SD) | 18.64 (3.34) | 19.37 (3.82) | 19.52 (3.89) | 20.83 (3.77) |
| Life satisfaction, M (SD) | 6.03 (1.84) | 6.18 (1.80) | 6.44 (1.86) | 6.98 (2.11) |
| Withdrawal symptoms, M (SD) | 3.69 (1.22) | 3.51 (1.21) | 3.06 (1.18) | 3.01 (1.24) |
| Acceptability |  |  |  |  |
| Total score^3^, M (SD) | 3.86 (0.66) | 3.63 (0.74) | 3.61 (0.87) | 3.54 (0.97) |
| Usefulness in quitting smoking 1, M (SD) | 3.92 (1.07) | 3.42 (1.09) | 2.38 (1.05) | 3.65 (1.15) |
| Usefulness in quitting smoking 2, M (SD) | 3.37 (0.93) | 3.32 (0.88) | 3.21 (0.97) | 3.51 (1.17) |
| Satisfaction with the program 1, M (SD) | 3.88 (0.68) | 3.78 (0.81) | 3.79 (0.75) | 3.93 (0.85) |
| Satisfaction with the program 2, M (SD) | 7.37 (2.30) | 6.8 (2.59) | 6.86 (2.98) | 6.89 (2.95) |
| Informativeness of the content, M (SD) | 4.44 (0.61) | 4.29 (0.67) | 4.29 (0.89) | 4.29 (0.9) |

**Note.** PPA = Point prevalence abstinence, CCA = Complete cases analysis, ITT = Intention-to-treat

^1,2^ Only participants who self-reported 7-day PPA were asked to answer this question

^3^ For the calculation of the total acceptability score, item “Satisfaction with the program 2” (range 0-10) was z-standardized to match the other items (range 1-5)

**Table A 4** Other outcome at 4 weeks, 8 weeks, 12 weeks, and 24 weeks follow-ups (N = 71)

| Other variables | 4 weeks post-baseline (t_1_)  n = 66 | 8 weeks post-baseline (t_2_)  n = 62 | 12 weeks post-baseline (t_3_)  n = 63 | 24 weeks post-baseline (t_4_)  n = 54 |
| --- | --- | --- | --- | --- |
| **Adherence and engagement** | | | | |
| Use of nuumi EC in the past 7 days | CCA: 89.4% (59/66)  ITT: 83.1% (59/71) | CCA: 75.8% (47/62)  ITT: 66.2% (47/71) | CCA: 60.3% (38/63)  ITT: 53.5% (38/71) | CCA: 27.8% (15/54)  ITT: 21.1% (15/71) |
| Use of nuumi EC in cigarette abstinent individuals^1^ | 95.6% (23/24) | 76.9% (20/26) | 60.7% (17/28) | 26.1% (6/23) |
| Use of nuumi EC in non-cigarette abstinent individuals^2^ | 85.7% (36/42) | 75.0% (27/36) | 60.0% (21/35) | 32.1% (9/28) |
| Days nuumi EC used, M (SD) | 6.12 (1.60) | 5.98 (1.70) | 5.00 (2.42) | 4.47 (2.36) |
| Engagement with nuumi app |  |  |  |  |
| Number of days used per week, M (SD) | 5.58 (2.06) | 4.21 (2.46) | 2.81 (2.62) | 0.94 (1.57) |
| Modules completed, M (SD) | 2.67 (1.38) | 3.77 (2.52) | 4.63 (2.81) | 4.89 (3.06) |
| Minutes meditated, M (SD) | 12.33 (15.92) | 30.82 (59.33) | 34.85 (66.93) | 46.98 (112.79) |
| **Subjective evaluation of the nuumi EC and side effects** | | | | |
| Usefulness in abstaining from cigarettes, M (SD) | 3.92 (1.07) | 3.81 (1.14) | 3.71 (1.17) | 3.59 (1.17) |
| Subjective evaluation total score, M (SD) | 2.19 (0.51) | 2.2 (0.51) | 2.15 (0.51) | 2.06 (0.47) |
| Side effects total score, M (SD) | 0.73 (0.44) | 0.76 (0.58) | 0.52 (0.48) | 0.61 (0.62) |
| **Other smoking-behavior related variables** | | | | |
| Use of other ECs | 10.6% (7/66) | 9.7% (6/62) | 14.3% (9/63) | 11.1% (6/54) |
| Current participation in other smoking cessation program | 1.5% (1/66) | 0% (0/62) | 0% (0/63) | 0% (0/54) |
| Current use of NRT | 0% (0/66) | 0% (0/62) | 0% (0/63) | 1.9% (1/54) |
| Dependence on nuumi EC, M (SD) | 7.46 (3.83) | 7.57 (4.21) | 7.37 (3.89) | 4.4 (3.58) |
| No dependence^3^ | 18.6% (11/59) | 17.0% (8/47) | 23.6% (9/38) | 40.0% (6/15) |
| Low dependence^3^ | 33.9% (20/59) | 42.6% (20/47) | 36.8% (14/38) | 40.0% (6/15) |
| Medium dependence^3^ | 39.0% (23/59) | 29.8% (14/47) | 31.6% (12/38) | 20.0% (3/15) |
| High dependence^3^ | 8.5% (5/59) | 10.6% (5/47) | 7.9% (3/38) | 0% (0/15) |

*Note.*

^1^ The denominator reflects individuals who self-reported 7-day PPA at the respective follow-up

^2^ The denominator reflects individuals who did not self-reported 7-day PPA at the respective follow-up

^3^ The denominator reflects individuals who self-reported having used the nuumi EC in the past 7 days at the respective follow-up
